# Integrating plasma protein-centric multi-omics to identify potential therapeutic targets for pancreatic cancer

**DOI:** 10.1186/s12967-024-05363-9

**Published:** 2024-06-10

**Authors:** Siyu Zhou, Baian Tao, Yujie Guo, Jichun Gu, Hengchao Li, Caifeng Zou, Sichong Tang, Shuheng Jiang, Deliang Fu, Ji Li

**Affiliations:** 1 Department of Pancreatic Surgery, Huashan Hospital, Fudan University, Shanghai, 200040 China; 2 School of Medicine, Fudan University, Shanghai, 200240 China; 3 State Key Laboratory of Oncogenes and Related Genes, Shanghai Cancer Institute, Renji Hospital, School of Medicine, Shanghai Jiao Tong University, Shanghai, 200240 China

**Keywords:** Pancreatic cancer, Plasma proteome, Therapeutic target, Mendelian randomization

## Abstract

**Background:**

Deciphering the role of plasma proteins in pancreatic cancer (PC) susceptibility can aid in identifying novel targets for diagnosis and treatment.

**Methods:**

We examined the relationship between genetically determined levels of plasma proteins and PC through a systemic proteome-wide Mendelian randomization (MR) analysis utilizing cis-pQTLs from multiple centers. Rigorous sensitivity analyses, colocalization, reverse MR, replications with varying instrumental variable selections and additional datasets, as well as subsequent meta-analysis, were utilized to confirm the robustness of significant findings. The causative effect of corresponding protein-coding genes’ expression and their expression pattern in single-cell types were then investigated. Enrichment analysis, between-protein interaction and causation, knock-out mice models, and mediation analysis with established PC risk factors were applied to indicate the pathogenetic pathways. These candidate targets were ultimately prioritized upon druggability and potential side effects predicted by a phenome-wide MR.

**Results:**

Twenty-one PC-related circulating proteins were identified in the exploratory phase with no evidence for horizontal pleiotropy or reverse causation. Of these, 11 were confirmed in a meta-analysis integrating external validations. The causality at a transcription level was repeated for neutrophil elastase, hydroxyacylglutathione hydrolase, lipase member N, protein disulfide-isomerase A5, xyloside xylosyltransferase 1. The carbohydrate sulfotransferase 11 and histo-blood group ABO system transferase exhibited high-support genetic colocalization evidence and were found to affect PC carcinogenesis partially through modulating body mass index and type 2 diabetes, respectively. Approved drugs have been established for eight candidate targets, which could potentially be repurposed for PC therapies. The phenome-wide investigation revealed 12 proteins associated with 51 non-PC traits, and interference on protein disulfide-isomerase A5 and cystatin-D would increase the risk of other malignancies.

**Conclusions:**

By employing comprehensive methodologies, this study demonstrated a genetic predisposition linking 21 circulating proteins to PC risk. Our findings shed new light on the PC etiology and highlighted potential targets as priorities for future efforts in early diagnosis and therapeutic strategies of PC.

**Supplementary Information:**

The online version contains supplementary material available at 10.1186/s12967-024-05363-9.

## Introduction

Pancreatic cancer (PC) is one of the leading causes to cancer death worldwide with increasing incidence and a meager 5-year survival rate of less than 9% [1, 2]. Approximately 80% of patients present with advanced and unresectable disease at diagnosis, which is partially attributed to the asymptomatic nature and difficulty in early detection [3]. Precancerous or early-stage lesions cannot be efficiently recognized merely by imaging alterations, implying the significance of exploring reliable diagnostic biomarkers [4]. But even for resectable PC, the prognosis of patients is optimistic as a result of rapid postoperation relapse and chemotherapy resistance [5]. Thus, novel available therapeutic strategies are warranted.

The plasma proteins as vital components in circulating blood, produced by cellular leakage and active secretion, are involved in various crucial physiological and pathological processes, and can thereby act as a reflection of the overall physical condition as well as possible druggable targets for illnesses [6–8]. Specifically, several circulating proteins are suggested to be biomarkers for inflammation, infection, and some systemic diseases [9–11]. With regard to malignancies, a number of cross-sectional studies have looked into the discrepancy in circulating protein levels between cancer sufferers and healthy controls in an attempt to establish the intricate protein-carcinogenesis connection [12–15]. But the nature of their observational studies restricts the reliability of conclusions due to potential confounding bias and reverse causation [16].

Recently, a series of large-scale proteomic research have identified the protein quantitative trait loci (pQTLs), enabling the causality inference for the effect of plasma protein on PC susceptibility via a two-sample Mendelian Randomization (MR) method, which utilizes genetic variants as instrumental variables to mimic randomized controlled trials [17–19]. Since MR results are less likely to be biased by confounders and reverse causation, the MR method is widely applied in investigating the causative factors for outcomes, such as the causal correlation of peripheral metabolites or gut microbiome with PC [20–22]. Of note, as the extension of MR methodology, proteome-wide MR studies focus on the genetic-determined circulating protein concentration and disease etiology and have been employed for exploring carcinoma-related biomarkers or promising interference targets in tumors like colorectal cancer, breast cancer, and lung cancer [23–26].

In the present study, we integrated cis-pQTL data for a proteome-wide MR analysis to identify PC-associated plasma proteins. Bidirectional MR, replicative validation and meta-analysis, and Bayesian colocalization were used to confirm the primary results. Then the corresponding protein-coding gene expressions were also analyzed regarding their causal effect on PC and their expression pattern in single-cell types. The function and involved pathways of these targets were preliminarily investigated through enrichment analysis, knock-out mice models, and between-protein interaction and causation. The interplay network between circulating proteins, known PC risk factors, and PC was further analyzed and discussed. Finally, drug-target databases were inquired to prioritize the druggable targets, and a phenome-wide MR was conducted to evaluate the drug safety and repurposing.

## Methods

### Overall design

**Fig. 1:**
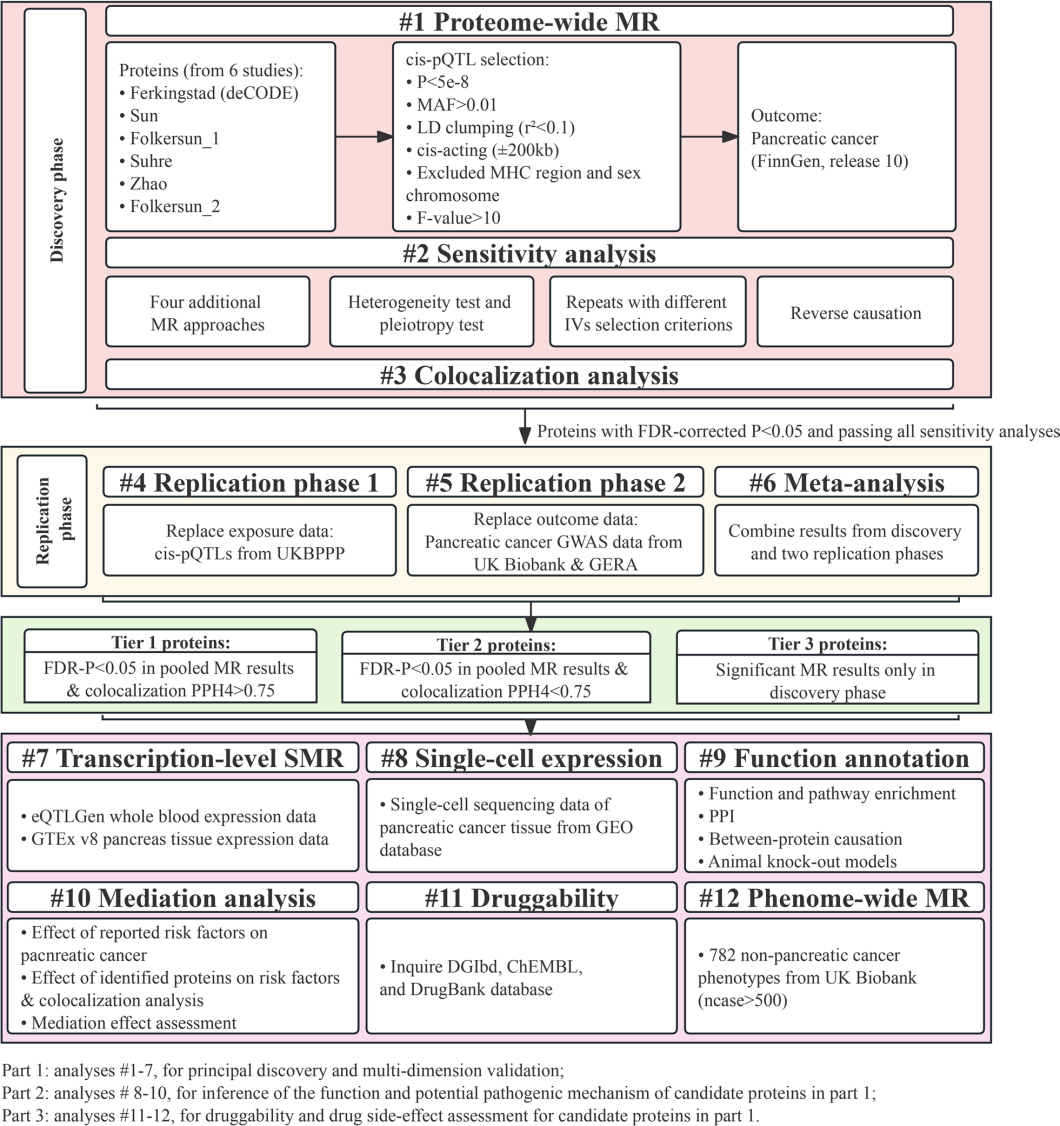
Flowchart of the study design. MR, Mendelian randomization; IV, instrumental variable; pQTL, protein quantitative trait loci; FDR, false discovery rate; UKBPPP, UK Biobank Pharma Proteomics Project; GWAS, genome-wide association study; GERA, Kaiser Permanente Genetic Epidemiology Research on Adult Health and Aging cohort; GTEx v8, Genotype-Tissue Expression Project version 8; GEO, Gene Expression Omnibus; PPI, protein-protein interaction; DGIdb, Drug Gene Interaction Database

The workflow and methodology of this study are outlined in Fig. 1. In brief, cis-pQTL data derived from six publicly accessible datasets were employed to conduct a proteome-wide, two-sample MR in the primary study phase. Subsequently, a three-part analytic protocol was applied to enhance and expand our initial findings. For part one, we employed several sensitivity analyses, bidirectional MR, Bayesian colocalizaion, external replications and meta-analysis, and replicative MR analysis in transcription level to validate the primary proteome-wide MR results. For part two, single-cell type expression analysis, GO/KEGG enrichment, mutual causality, protein–protein interaction (PPI) network, single gene knock-out mice models, and mediation analysis, were used to annotate the function and infer the potential pathogenic pathways of these PC-associated candidates obtained from analysis in part one. For part three, the druggability and possible side effects estimated by a phenome-wide MR were assessed for prioritizing these therapeutic targets. The P-values were all adjusted by the Benjamini–Hochberg false discovery rate (FDR) in multiple tests in our study.

### Study datasets and genetic instruments selection

In the exploratory phase, genome-wide association study (GWAS) summary statistics regarding PC were acquired from the FinnGen consortium R10 release (https://www.finngen.fi/en) [27]. In the present research, PC was defined as malignant neoplasm of the pancreas, incorporating pancreatic ductal adenocarcinoma and other pathological types of malignant pancreatic tumors. Summary statistics of genetic associations with circulating proteins were obtained from six distinct large-scale proteomic studies (Ferkingstad et al., 4907 proteins; Sun et al., 3282 proteins; Folkersen_1., 82 proteins; Suhre., 1124 proteins; Folkersen_2., 90 proteins; Zhao., 91 proteins [6, 28–32]). Detailed descriptions of the above datasets can be found in original publications. We harmonized these proteomic data by remapping protein IDs onto corresponding gene symbols. For proteins presented in multiple datasets, or those with different probes or isoforms, we calculated the proportion of variability (R2) (see below), and the one with the largest R2 was retained. To satisfy the basic assumptions of MR, we filtered the extracted pQTLs upon the following criterion: (1) pQTLs with genome-wide significant (P < 5E-8) association with any protein; (2) minor allele frequency (MAF) > 0.01; (3) linkage disequilibrium (LD) R2 between SNPs was controlled < 0.1 within 10 Mb; (4) cis-pQTL was defined as pQLT being cis-acting (within 200 kb upstream or downstream of the protein-coding gene region); (5) SNPs located in the major histocompatibility complex (MHC) region (chromosome 6, 31-33 Mb) and sex chromosome were excluded; (6) R2 and F-value were computed to evaluate the strength of instrumental variables (IVs) (R2 = 2*MAF*(1-MAF)*beta*beta; F-value = R2*(N-2)/(1-R2)) [33], and SNPs with F-value < 10 were removed; (7) cis-pQTL should not be directly associated with PC (P-value > 1E-5). The involved GWAS studies mainly enrolled participants of European ancestry and had all received approval from their corresponding ethical review committees.

### Proteome-wide MR, sensitivity analysis, and reverse MR analysis

The “TwoSampleMR” R package was utilized for a proteome-wide MR analysis [34]. The MR methodology employed SNPs as IVs to infer causal relationship between two traits, and the wald ratio (No.SNPs = 1) and inverse variance-weighted (IVW) algorithms (No.SNPs > 1) were applied as principal MR approaches since they were most efficient when all IVs were validsince [35]. The wald ratio algorithm calculated the effect ratio of one variant in exposure and outcome. When there was at least two instruments, the IVW algorithm was used to combines the ratio estimates of each variant in a meta-analysis model. Before MR analysis was performed, we harmonized the exposure and outcome data using the “harmonise_data” function. This process extracted IVs that overlapped in the filtered exposure data with the outcome data and automatically removed incompatible and palindromic SNPs. The presence of heterogeneity was assessed using Cochrane’s Q test, and a test P-value less than 0.05 indicated heterogeneous IVs. In this case, a random-effect IVW model would be used. Otherwise, a fixed-effect IVW MR was performed. The P-values of the proteome-wide MR results were corrected with the Benjamini–Hochberg FDR method, and causal associations with FDR-corrected P-values less than 0.05 were considered significant. Additionally, the MR-Egger regression intercept test, MR-Pleiotropy Residual Sum and Outlier (MR-PRESSO) methodology, and MR-PRESSO global test were employed to evaluate the horizontal pleiotropy [36, 37]. Subsequently, MR analyses with four additional approaches including weighted median, MR-Egger, weighted mode, and simple mode were performed as part of sensitivity analyses. Since the MR results were potentially susceptible to the IVs selection, we re-analyze the data after modifying the IVs inclusion criterion by taking the parameter of LD R2 threshold of 0.001, 0.01, 0.2, and 0.3, respectively. Additionally, the presence of reverse causation was assessed by an inverse MR. However, only two SNPs were initially extracted as IVs proxied for PC after pruning instruments with a stringent threshold for P-value (P-value < 5E-8) and LD clumping (r^2^ = 0.001). Thus, a broader threshold for the P-value of 5E-6 was adopted as a replicative validation in the reverse MR. Upon integrating results of all above sensitivity analyses, potential targets with robust evidence in the discovery phase were defined as: (1) significantly associated with PC after multiple tests correction; (2) no pleiotropic outliers detected, or significant MR results in the re-analysis after removing outliers; (3) absence of horizontal pleiotropy revealed by Egger intercept or MR-PRESSO global test; (4) identical effect direction to primary results in sensitivity analyses using additional MR approaches and varying LD parameters; (5) no evidence showing reverse causation.

### Bayesian colocalization

Circulating proteins in significant relation to PC and passing all sensitivity tests were analyzed with Bayesian colocalization using “coloc” R package to illuminate whether a protein and PC were linked to a shared causal variant or the association was driven by the confounding of linkage disequilibrium [38]. Aligning with previous studies, we adopted default parameters of p1 = 1E-4, p2 = 1E-4, and p12 = 1E-5 in this process [39]. P1 and p2 represented the prior probability that a SNP is significantly correlated with protein and PC risk, respectively, and p12 represented the prior probability of a SNP being associated with both traits. For each locus, the posterior probabilities of the following five hypotheses were assessed: (1) H0: no causal variant for either plasma protein or PC; (2) H1: one causal variant only for protein; (3) H2: one causal variant only for PC; (4) H3: two different causal variants for protein and PC respectively; (5) H4 one shared causal variant for both PC and plasma protein. High-support evidence of colocalization was considered in cases with the posterior probabilities of H4 (PPH4) over 0.75, and medium-support evidence of colocalization was defined as PPH4 less than 0.75 but greater than 0.5 [40].

### Replication and meta-analysis

We repeated our primary analysis in a two-phase validation. In replication phase 1, cis-pQTLs were obtained from a plasma proteomic association study in UK Biobank [41], which incorporated 2923 proteins and 54,219 European ancestry individuals, while in replication phase 2, the genome-wide association data for PC was replaced by an integrated GWAS study involving population from both UK Biobank and the Kaiser Permanente Genetic Epidemiology Research on Adult Health and Aging cohort (GERA) with a sample size of 411,013 [42]. All of the above datasets were essentially GWAS data of the same phenotype with that of the exposure or outcome in the discovery phase. They had adequate sample size and large number of measured SNPs. These GWAS studies all incorporated European populations, and there was no population overlap in each exposure-outcome pair because they were from distinct research cohorts. So the selection for above validation datasets met the requirements of MR analysis and ensured these data were suitable to be employed in further replicative phases. The cis-pQTL filtering process was identical to that of the discovery phase. and sensitivity analyses (heterogeneity test, pleiotropy test, and supplementary MR approaches) were performed as usual. Moreover, to expand our findings, proteins without significant links to PC in the primary stage were also analyzed using alternative data sources. Finally, a meta-analysis was conducted to combine MR estimates from the discovery phase and two replication phases, which would be deemed as the ultimate results of external validation. Heterogeneity of meta-analysis was assessed by the statistics I2 to determine the use of random effect or fixed effect models [43]. We then categorized the identified PC-related candidates that passed all sensitivity analyses in the primary stage into three tiers according to the evidentiary strength of colocalization and external validation: (1) tier 1 proteins: FDR-corrected P-value < 0.05 in meta-analysis, consistent effect direction in discovery and validation phases, and colocalization PPH4 > 0.75; (2) tier 2 proteins: FDR-corrected P-value < 0.05 in meta-analysis, consistent effect direction in discovery and validation phases, and colocalization PPH4 < 0.75; (3) tier 3 proteins: unsuccessful replication in external validation.

### Transcriptome-level MR and SMR analysis

For the sake of further investigating the causation of the corresponding protein-coding genes’ expression on PC, we obtained full expression quantitative trait loci (eQTL) data for whole blood tissue from the eQTLGen Consortium (https://eqtlgen.org/), which comprised genetic associations with the expression of 16,987 genes among 31,684 mostly healthy participants [44]. The selection standard for cis-eQTLs was the same as that of cis-pQTLs (see above), and the acquired cis-eQTLs for candidate targets were then employed in the subsequent transcription-level two-sample MR analysis. In addition, the summary-data-based MR (SMR) test using the top hit eQTL as instrument was also implemented with SMR software (SMR v1.3.1) as a sensitivity analysis. And the heterogeneity in independent instrument (HEIDI) was conducted to distinguish the identified relationships from pleiotropy and genetic linkage [45]. The SMR-formatted cis-eQTLs data could be accessed from publicly available link (https://molgenis26.gcc.rug.nl/downloads/eqtlgen/cis-eqtl/SMR_formatted/cis-eQTL-SMR_20191212.tar.gz). Likewise, the SMR and HEIDI tests were also performed to explore the gene-PC association in pancreas tissue by utilizing the SMR-formatted cis-eQTLs data acquired from https://yanglab.westlake.edu.cn/software/smr/#DataResource. Results would be considered positive and valid when the P-value for SMR was less than 0.05 and the P-value for HEIDI test was over 0.05.

### Single cell-type expression analysis

We downloaded the single-cell RNA sequencing (scRNA-seq) data of target protein-coding genes in 16 PC samples from the Gene Expression Omnibus (GEO) database (Registration number: GSE155698) [46]. The scRNA-seq data was then processed with “Seurat” R package [47]. The created and merged Seurat object incorporated 49,333 cells and 32,738 features. Firstly, in order to obtain high-quality data on single-cell RNA expression, the following filtering standard was established: 1. exclusion of genes with expression lower than five counts in one cell; 2. exclusion of cells with < 300 or > 4000 measured genes; 3. exclusion of cells with > 10% mitochondrial contamination. As a consequence, a total of 28,994 high-quality cells and 23,384 features were remaining for further analysis. Data after quality control would be normalized with “NormalizeData” function, which was used to normalize raw read counts by applying a scale factor of 10,000 and logarithmically transforming the values to stabilize the variance across genes with different expression levels. Subsequently the “SingleR” R package was employed for cell clusters annotation, which assigned cell identities by correlating single-cell RNA expression profiles with reference datasets of known cell types, enabling precise identification and analysis of distinct cell populations [48]. To illustrate whether the expression of the target gene was enriched in a specific cell cluster, differential gene expression across cell types was analyzed using the Wilcoxon Rank Sum test, and the enrichment would be defined as significant when FDR-corrected P-value < 0.05 and |Log2(fold-change)|> 1.

### Function and pathway enrichment

For exploring the potential biological implication of identified PC-related circulating proteins, enrichment analysis with regard to the GO function terms (biological processes, cellular components, and molecular functions) and Kyoto Encyclopedia of Genes and Genomes (KEGG) pathways was applied [49]. This enrichment methodology was conducted by evaluating whether the presence of specific genes within a given pathway significantly exceeded what would be anticipated by chance, as determined by the proportion of genes in the background dataset associated with that pathway. Functions or pathways with FDR-corrected P-value less than 0.05 were deemed significantly enriched.

### Protein–protein interaction (PPI) and mutual causation

The protein–protein interaction (PPI) network was constructed with the Search Tool for the Retrieval of Interacting Genes (STRING) database (https://string-db.org) with a minimum interaction confidence score of 0.4. To further investigate the interplay between circulating protein levels, we carried out a series of two-sample MR analyses pair by pair of those candidate target proteins with each other.

### Animal knock-out models

For circulating proteins shown as potential therapeutic targets, we queried the single gene knock-out mice models through the Mouse Genome Informatics (MGI) website (http://www.informatics.jax.org) to verify their biological function, as well as the possible side effects that might be brought about by the targeted therapy. Phenotypes in relation to single gene knock-out were manually classified and displayed in three categories: neoplastic phenotypes, phenotypes of the digestive system, and phenotypes of others.

### Mediation analysis

The etiology of malignant tumors was complicated, and the effect of proteins on tumorigenesis might not be direct but indirect through established risk factors. In order to validate the above hypothesis, we designed a four-step analysis protocol: (1) identify risk factors for PC through previous literature; (2) verify the causation of these risk factors on PC via a two-sample MR (Effect1); (3) compute the MR estimate of PC-associated circulating proteins on these risk factors (Effect2) and conduct colocalization analysis; (4) estimate the mediation effect and direct effect. The total effect from protein to PC was equal to the MR estimate obtained from the proteome-wide MR analysis of the discovery phase, and the mediation/indirect effect was calculated as (Effect1 * Effect2), while the direct effect was calculated as (Effect_total_—Effect_mediation_) [50]. There would be a possibility that the mediation effect existed if the effect direction of all causal pairs in the association between proteins, PC, and risk factors followed right logic. The confidence interval (CI) and P-value of mediating effect were estimated by the delta method.

### Druggability assessment and phenome-wide MR analysis

Three drug-targets databases (Drugbank: https://go.drugbank.com/; ChEMBL: https://www.ebi.ac.uk/chembl/; DGIdb: https://www.dgidb.org/) were queried to identify available drugs targeting the candidate circulating proteins. Details of the drugs and drug-gene interaction were documented. Furthermore, a phenome-wide MR analysis was performed to appraise the drug safety and repurposing. Summary statistics of genetic association with extensive phenotypes were collected from UK Biobank (https://pheweb.org/UKB-SAIGE/), and only traits with cases over 500 were retained as outcomes in the phenome-wide MR. All the cis-pQTLs proxied for plasma proteins and all the parameters used in MR process were identical to that of the discovery phase.

## Results

### Proteome-wide MR identified 21 plasma proteins causally affecting PC susceptibility in the discovery phase

In the discovery phase, after removing SNPs with P-value > 5E-8 or MAF < 0.01, IVs were available for 4,790, 2,229, 63, 356, 85, and 75 proteins in the proteomic studies of Ferkingstad et al., Sun et al., Folkersen_1., Suhre., Folkersen_2., and Zhao., respectively. After LD clumping and removal of pQTLs located in sex chromosome or MHC region and pQLTs located away from protein-coding gene region, 1,774, 658, 32, 228, 72, and 61 proteins were retained respectively. After deletion of IVs with F-value < 10 and harmonization, a total of 28,050 SNPs were available to proxy 2,781 proteins. Then, proteins that appeared in more than one dataset were eliminated and the one with the largest R2 sum was retained. Finally, a total of 19,379 SNPs were applied as IVs for 1,751 proteins in the primary MR analysis. The median number of cis-pQTLs used for proxying single protein was six (ranging from one to 90). And instruments for most of the proteins (1372/1751) were derived from the study of Ferkingstad et al. [28], according to their R2 sum.

**Fig. 2:**
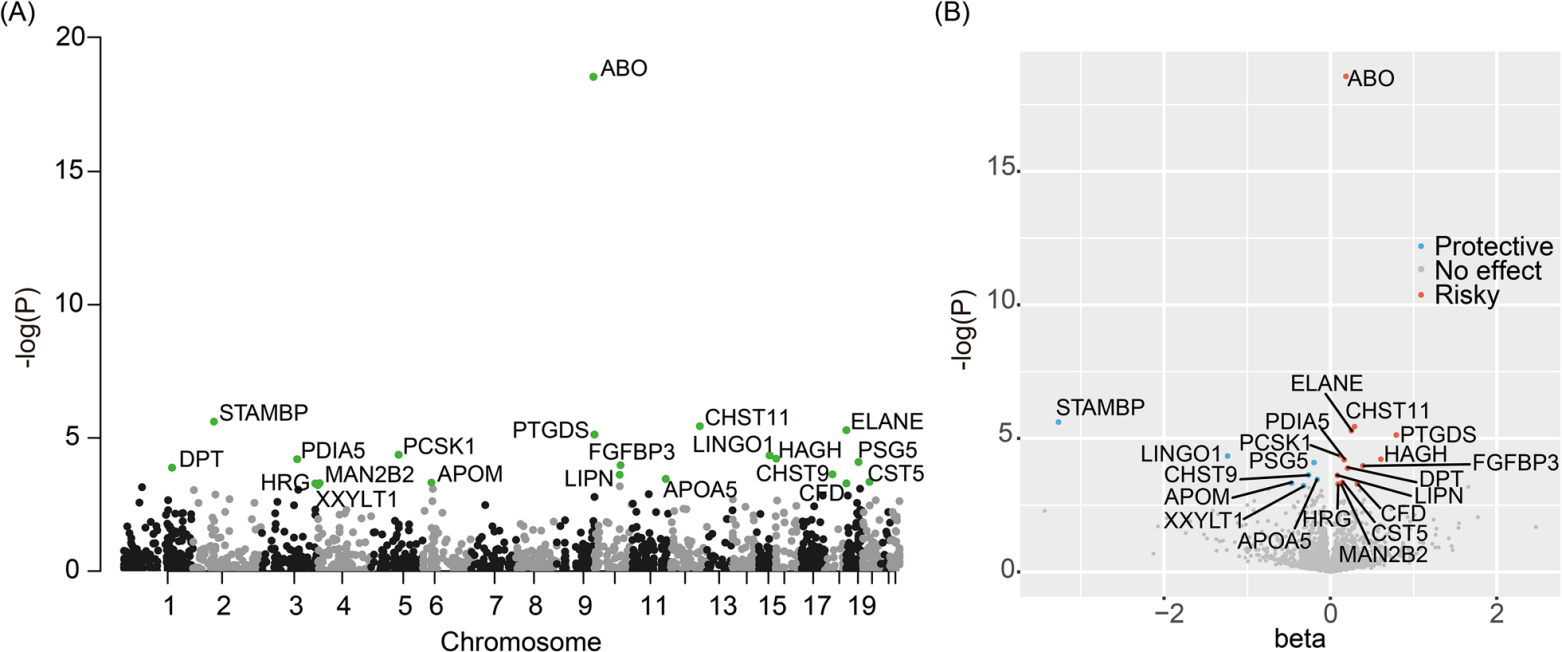
The Manhattan plot (**A**) and volcano plot (**B**) of the proteome-wide Mendelian randomization analysis in the discovery phase

The primary results revealed 21 significant protein-PC association pairs after correcting P-values with FDR (FDR-corrected P-value < 0.05), as displayed in Fig. 2 in the form of a Manhattan map and volcano plot. In the sensitivity analysis with additional MR approaches (MR Egger, weighted median, weighted mode, and simple mode), the direction of causality of significant findings were all in concordance with the primary conclusions (Additional file1: Table S1). In addition, no inter-SNPs heterogeneity was found via Cochran’s Q test, and no horizontal pleiotropy was observed through the Egger intercept test and the MR-PRESSO global test for these positive MR results. Then we altered the clumping parameter (LD R2 = 0.001, 0.01, 0.2, and 0.3, respectively) in four separate repeats for the 21 identified proteins. Of the 84 replicative inspections, none of them demonstrated an effect direction contrary to the original estimates, and the majority of the MR analyses (74/84) still yield a statistically significant association (Additional file1: Table S2). In terms of the reverse causation, no obvious impact from PC on circulating protein concentrations was found after multiple testing corrections, whenever the IVs for PC were selected based on a strict (5E-8) or relatively loosen (5E-6) P-value threshold (Additional file1: Table S3). Collectively, the associations between PC and 21 genetically predicted protein levels passed all of the sensitivity analyses, implicating robust evidence.

Of the 21 PC-associated circulating proteins, 14 proteins exhibited tumor-promoting efficacy, including ABO (Histo-blood group ABO system transferase), PTGDS (Prostaglandin-H2 D-isomerase), CFD (Complement factor D), CHST11 (Carbohydrate sulfotransferase 11), ELANE (Neutrophil elastase), HAGH (Hydroxyacylglutathione hydrolase, mitochondrial), CST5 (Cystatin-D), PCSK1 (Neuroendocrine convertase 1), HRG (Histidine-rich glycoprotein), LIPN (Lipase member N), MAN2B2 (Epididymis-specific alpha-mannosidase), DPT (Dermatopontin), PDIA5 (Protein disulfide-isomerase A5), and FGFBP3 (Fibroblast growth factor-binding protein 3). Among these candidates, ABO yielded the most prominent causality (OR (95%CI) = 1.21 (1.16–1.26), FDR-corrected P-value = 4.88E-16).

The other 7 plasma proteins displayed a protective action against PC onset, including CHST9 (Carbohydrate sulfotransferase 9), PSG5 (Pregnancy-specific beta-1-glycoprotein 5), STAMBP (STAM-binding protein), LINGO1 (Leucine-rich repeat and immunoglobulin-like domain-containing nogo receptor-interacting protein 1), APOA5 (Apolipoprotein A-V), APOM (Apolipoprotein M), and XXYLT1 (Xyloside xylosyltransferase 1), among which the STAMBP showed the most significant negative causation (OR (95%CI) = 0.04 (0.01–0.15), FDR-corrected P-value = 2.12E-3).

### Colocalization analysis

To distinguish the causal relationship between genetically determined circulating protein levels and PC from linkage disequilibrium, the colocalization analysis was applied. Among the 21 candidate proteins, the PPH4 values for ABO (PPH4 = 0.967) and CHST11 (PPH4 = 0.757) were over 0.75, suggesting high-support evidence for an association linked to a shared causal variant (Additional file1: Table S4). Besides, medium-level evidence of colocalization (PPH4 > 0.5) was observed linking LINGO1 (PPH4 = 0.638) and PTGDS (PPH4 = 0.619) to PC. As for the rest of the 17 circulating proteins, no significant satisfaction for assumption H4 was revealed. However, it was worth noting that negative results did not inherently invalidate the findings obtained from MR [51].

### Replication and meta-analysis

For the prominent associations found in the discovery stage, we repeated the analysis with additional data sources to inspect the robustness of conclusions. In replication phase 1, when the GWAS data for PC was obtained from a study integrating UK Biobank and GERA participants, the genetically predicted circulating level of ABO and HRG had a significant causal link with PC. Furthermore, for proteins with negative results in the primary phase, replicative proteome-wide analysis was still conducted to expand our findings. As demonstrated in Additional file1: Table S5, EVL, PCSK9, NPTX2, and GPC1 were additionally identified with potential influence on PC occurrence in replication phase 1 after multiple testing corrections. In replication phase 2, cis-pQTLs extracted from UK Biobank consortium were available for 11 out of the 21 identified plasma proteins, and 8 out of the 11 available proteins (ABO, CFD, CST5, DPT, FGFBP3, HRG, PDIA5, PTGDS) were still causally associated with PC risk. Likewise, proteome-wide MR analyses investigating 1,939 proteins from the UK Biobank Pharma Proteomics Project (UKBPPP) study were also performed in replication phase 2, whose full results can be accessed in Additional file1: Table S6. Subsequently, a meta-analysis was employed to integrate MR estimates from both the discovery and replication phases. After pooling effects and correcting P-values with FDR method, statistically significant associations with PC were observed in 11 proteins including ABO, CFD, CHST11, CHST9, CST5, ELANE, HAGH, HRG, LIPN, MAN2B2, PCSK1, PSG5, and PTGDS (Additional file1: Table S7). However, of these candidates, inconsistent causation direction between the discovery and validation phases was observed for PTGDS and MAN2B2. Accordingly, taking all the above results together, the circulating proteins were grouped into three categories (see methods). ABO and CHST11 lay in the top tier with the strongest evidence from both colocalization and replications. The tier 2 proteins incorporated proteins with successful repeats in external validation but no colocalization evidence, including CFD, ELANE, HAGH, CST5, CHST9, PSG5, PCSK1, HRG, and LIPN. And the rest of the 10 proteins were classified as tier 3 category including PTGDS, MAN2B2, DPT, PDIA5, STAMBP, FGFBP3, LINGO1, APOA5, APOM, and XXYLT1. Detailed information for protein categorizing was summarized in Table 1.

**Table 1:** Summary results of the primary MR analysis, meta-analysis, colocalization, and protein classification

Protein	Full protein name	Primary analysis					Meta-analysis results combined with replications			Colocalization		Catagory
		OR (95%CI)	P-value	P_FDR_	Sensitivity analyses	Revers effect	OR (95%CI)	P-value	P_FDR_	PPH4	PPH3 + PPH4	
ABO	Histo-blood group ABO system transferase	1.21 (1.16–1.26)	2.790E-19	4.880E-16	Passed	No	1.22 (1.18–1.25)	1.350E-40	2.835E-39	0.967	1.000	Tier 1
CHST11	Carbohydrate sulfotransferase 11	1.34 (1.18–1.51)	3.630E-06	2.118E-03	Passed	No	1.28 (1.15–1.42)	5.320E-06	9.576E-05	0.757	0.853	Tier 1
CFD	Complement factor D	1.38 (1.15–1.66)	5.099E-04	4.512E-02	Passed	No	1.33 (1.18–1.51)	4.630E-06	8.797E-05	0.052	0.398	Tier 2
ELANE	Neutrophil elastase	1.29 (1.15–1.43)	5.130E-06	2.244E-03	Passed	No	1.27 (1.14–1.41)	5.890E-06	1.001E-04	0.217	0.503	Tier 2
HAGH	Hydroxyacylglutathione hydrolase, mitochondrial	1.83 (1.36–2.47)	6.040E-05	1.230E-02	Passed	No	1.70 (1.34–2.16)	1.080E-05	1.728E-04	0.254	0.385	Tier 2
CST5	Cystatin-D	1.15 (1.06–1.25)	4.487E-04	4.512E-02	Passed	No	1.11 (1.06–1.16)	1.110E-05	1.728E-04	0.044	0.211	Tier 2
CHST9	Carbohydrate sulfotransferase 9	0.77 (0.67–0.88)	2.340E-04	3.035E-02	Passed	No	0.77 (0.68–0.87)	4.330E-05	6.062E-04	0.202	0.302	Tier 2
PSG5	Pregnancy-specific beta-1-glycoprotein 5	0.82 (0.74–0.91)	8.060E-05	1.412E-02	Passed	No	0.83 (0.75–0.91)	5.260E-05	6.838E-04	0.410	0.865	Tier 2
PCSK1	Neuroendocrine convertase 1	1.12 (1.06–1.19)	4.300E-05	1.142E-02	Passed	No	1.11 (1.05–1.16)	1.121E-04	1.345E-03	0.413	0.468	Tier 2
HRG	Histidine-rich glycoprotein	1.10 (1.04–1.160)	5.154E-04	4.512E-02	Passed	No	1.14 (1.06–1.22)	2.301E-04	2.531E-03	0.066	0.197	Tier 2
LIPN	Lipase member N	1.09 (1.04–1.14)	2.426E-04	3.035E-02	Passed	No	1.07 (1.03–1.12)	3.437E-04	3.437E-03	0.252	0.316	Tier 2
PTGDS	Prostaglandin-H2 D-isomerase	2.21 (1.56–3.12)	7.500E-06	2.626E-03	Passed	No	2.04 (1.53–2.70)	9.100E-07	1.820E-05	0.619	0.690	Tier 3
MAN2B2	Epididymis-specific alpha-mannosidase	1.13 (1.06–1.22)	5.005E-04	4.512E-02	Passed	No	1.08 (1.03–1.13)	8.781E-04	7.903E-03	0.020	0.175	Tier 3
DPT	Dermatopontin	1.23 (1.11–1.36)	1.312E-04	1.915E-02	Passed	No	1.14 (0.99–1.30)	7.252E-02	5.802E-01	0.083	0.181	Tier 3
PDIA5	Protein disulfide-isomerase A5	1.18 (1.09–1.28)	6.320E-05	1.230E-02	Passed	No	1.14 (0.96–1.35)	1.308E-01	9.156E-01	0.139	0.285	Tier 3
STAMBP	STAM-binding protein	0.04 (0.01–0.15)	2.450E-06	2.118E-03	Passed	No	0.23 (0.03–1.75)	1.555E-01	9.330E-01	0.079	0.192	Tier 3
FGFBP3	Fibroblast growth factor-binding protein 3	1.48 (1.21–1.80)	1.062E-04	1.690E-02	Passed	No	1.26 (0.90–1.77)	1.762E-01	9.330E-01	0.414	0.788	Tier 3
LINGO1	Leucine-rich repeat and immunoglobulin-like domain-containing nogo receptor-interacting protein 1	0.29 (0.16–0.53)	4.570E-05	1.142E-02	Passed	No	0.76 (0.11–5.4)	7.840E-01	1.000E + 00	0.638	0.890	Tier 3
APOA5	Apolipoprotein A-V	0.85 (0.78–0.93)	3.479E-04	4.061E-02	Passed	No	0.92 (0.77–1.09)	3.356E-01	1.000E + 00	0.381	0.494	Tier 3
APOM	Apolipoprotein M	0.63 (0.48–0.81)	4.772E-04	4.512E-02	Passed	No	0.82 (0.46–1.48)	5.154E-01	1.000E + 00	0.472	0.585	Tier 3
XXYLT1	Xyloside xylosyltransferase 1	0.72 (0.60–0.87)	5.936E-04	4.949E-02	Passed	No	0.87 (0.58–1.31)	5.089E-01	1.000E + 00	0.085	0.302	Tier 3

PTGDS and MAN2B2 were categorized into tier 3 due to inconsistent effect direction in discovery and replication phase despite the significance in meta-analysis

MR, Mendelian randomization; OR, odds ratio; CI, confidence interval; FDR, false discovery rate; PPH, posterior probabilities of hypothesis

### Gene expression of candidate targets and PC risk

Among the 21 identified potential therapeutic targets, eQTLs for 13 corresponding protein-coding genes in whole blood tissues were finally acquired from eQTLGen consortium. In test with two-sample MR method, the association between PC and expression levels of ELANE, HAGH, LIPN, PDIA5, PTGDS, and XXYLT1 reached a statistical significance (Additional file1: Table S8). However, it was unfortunately discovered that the effect of PTGDS gene expression was opposite to its effect at a protein level. When applying SMR method using top hit eQTL and HEIDI test as supplementary sensitivity analyses, only STAMBP was identified to correlate with PC and pass the HEIDI test. In addition, eQTL data in pancreas tissues were only available for ABO, HAGH, and MAN2B2 from GTEx v8, and after employing these eQTLs in SMR-based analysis, ABO expression yielded a positive correlation with PC (P-value = 0.04), but the HEIDI test suggested a potential presence of heterogeneity (P-value for HEIDI = 0.004).

### Single-cell type expression in PC tissues

**Fig. 3:**
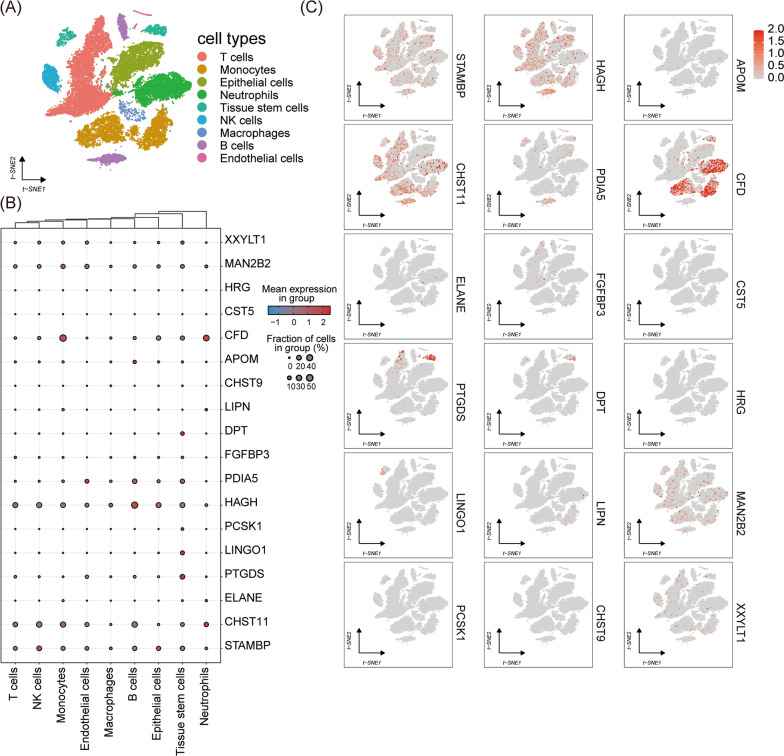
Single-cell type expression in pancreatic cancer tissue for the protein-coding genes of the identified candidate targets in the discovery proteome-wide Mendelian randomization investigation. **A** represented the nine cell clusters labeled and annotated by marker genes; **B** and **C** demonstrated the expression of candidate targets in each cell type

Single-cell type RNA sequencing data for 16 PC tissues were attracted from the dataset of GSE155698. Since ABO, PSG5, and APOA5 were not detected in the study, expressions of 18 protein-coding genes were then available for further analysis. As shown in Fig. 3A, after annotation for cell types, all cells were classified into nine clusters incorporating monocytes, T cells, epithelial cells, neutrophils, tissue stem cells, NK cells, macrophages, B cells, and endothelial cells. The cell type-specific gene expressions were demonstrated in Fig. 3B and C. Subsequently, in the Wilcoxon Rank Sum test for the differential gene expression across cell types, PTGDS was observed to be significantly enriched in tissue stem cells with FDP-corrected P-value < 0.05 and |log2(fold-change)|> 1, while CFD was enriched in immune cells such as monocytes, NK cells, and neutrophils.

### Pathway enrichment and protein interaction

**Fig. 4:**
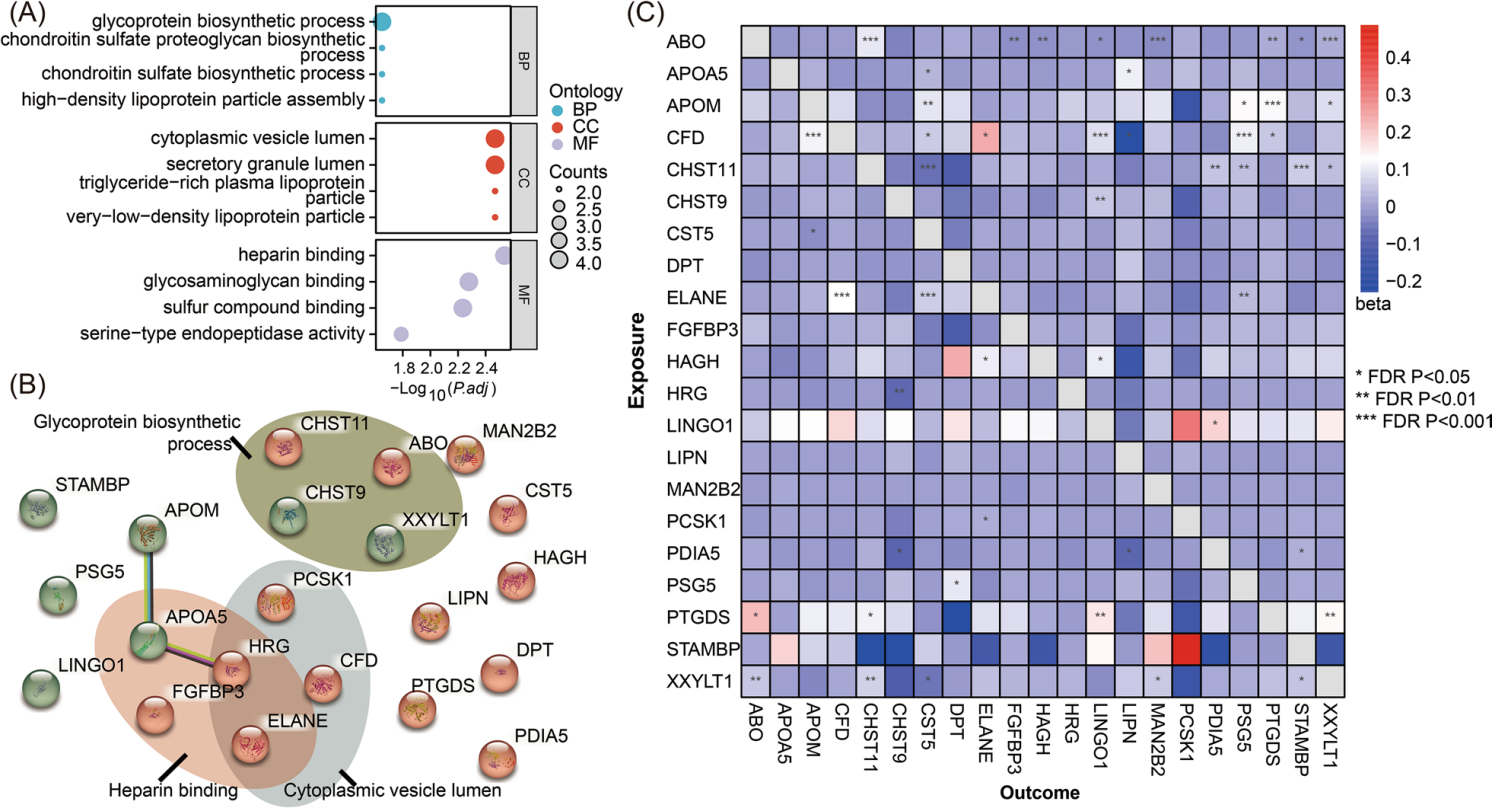
The functional enrichment (**A**), PPI (**B)**, and the between-protein causation analysis (**C**) of the identified 21 candidates in the discovery proteome-wide Mendelian randomization investigation. The red bubbles in PPI plot represent proteins acting to promote pancreatic cancer tumorigenesis, while green bubbles represent those acting to protect against pancreatic cancer. BP, biological procedure; CC, cellular component; MF, molecular function; PPI, protein–protein interaction

To explore whether these identified proteins were involved in a specific biological pathway, enrichment analysis for GO terms and KEGG pathways was performed. As displayed in Fig. 4A, the top enriched pathways for biological procedure (BP) included glycoprotein biosynthetic process, chondroitin sulfate proteoglycan biosynthetic process, chondroitin sulfate biosynthetic process, and high-density lipoprotein particle assembly. In terms of cellular component (CC), these proteins were involved in cytoplasmic vesicle lumen, secretory granule lumen, triglyceride-rich plasma lipoprotein particle, and very-low-density lipoprotein particle. Moreover, for the aspect of molecular function (MF), these targets were enriched in heparin binding, glycosaminoglycan binding, sulfur compound binding, and serine-type endopeptidase activity. However, no significantly enriched KEGG pathway was highlighted. PPI network analysis was used for investigating the interplay of the identified plasma proteins. A total of 17 interaction pairs involving 14 proteins were obtained, and when setting the threshold of confidence score as 0.4, only the interaction between APOM and APO5 (score = 0.942), and interaction between APO5 and HRG (score = 0.861) were observed (Fig. 4B). In addition, the mutual influence of the plasma level of these candidate proteins was inspected, and a total of 49 significant associations were identified (Fig. 4C). Of these, ABO was the one to have the greatest impact on the plasma level of other targets, and up to 8 circulating proteins (CHST11, XXYLT1, MAN2B2, FGFBP3, PTGDS, HAGH, LINGO1, STAMBP) were up- or down-regulated by ABO level.

### Single gene knock-out mouse models

The MGI resource was queried to identify the emerging phenotypes relevant to the knock-out of the potential targets. For the 21 potential target genes, among which 7 played protective roles against neoplasms, no new-onset tumor or other neoplastic trait was induced in mouse models merely by knocking out the single gene. With regard to digestive system-related traits, PCSK1 knock-out brought about chronic diarrhea and modification in intestinal goblet cells and enteroendocrine cells. Phenotypes of other systems produced by single gene knock-out could be accessed in Additional file 1: Table S9.

### Mediation analysis

Alcohol consumption, body mass index (BMI), smoking, chronic pancreatitis, and type 2 diabetes mellitus were identified as risk factors for PC through previous publications [52–56]. The two-sample MR method was applied to verify the correlation between risk factors and PC. As shown in Additional file 1: Table S10, BMI (P-value < 0.001) and type 2 diabetes mellitus (P-value = 0.021) significantly increased the risk of PC. However, the impact of alcohol consumption, smoking, and chronic pancreatitis on PC occurrence was not validated in our study (P-value > 0.05). In order to inspect whether the risk factors act as mediators in protein-PC connection, we analyzed the causal effect from the 21 candidate proteins to risk factors. After multiple testing corrections, several significant links were demonstrated: CHST11 and CHST9 were positively associated with PC; FGFBP3 and PCSK1 were negatively associated with smoking; FGFBP3 and MAN2B2 were negatively associated with type 2 diabetes mellitus while ABO was positively associated with it (Additional file1: Table S11). Considering the significance and the effect direction of above MR results, the indirect effect on PC onset was possibly valid only for ABO and CHST11, via the mediation of type 2 diabetes mellitus and BMI, respectively. The mediation effect was calculated as described in the method section, and the delta method was used to estimate the standard error. Therefore, the indirect effect of ABO on PC mediated by type 2 diabetes mellitus was 0.0049 (95%CI 0.0004–0.0094, P-value 0.035), and the corresponding mediation proportion was 2.61% (95%CI 0.19%-5.04%). The indirect effect of CHST11 on PC mediated by BMI was 0.0069 (95%CI 0.0014–0.0125, P-value = 0.015), and the corresponding mediation proportion was 2.40% (95%CI 0.47–4.33%).

### Druggability assessment

In druggability assessment, eight of 21 PC-associated plasma proteins, including ELANE, PSG5, HAGH, PCSK1, PTGDS, HRG, CHST11, and APO5, were revealed to be targeted for drug development (Additional file 1: Table S12). Drugs targeting ELANE have been applied in treatments for chronic obstructive pulmonary disorder (Alpha-1-proteinase inhibitor and Erdosteine) and neutropenia (Pegfilgrastim). Alitretinoin targeting PGS5 had been employed for topical treatment of cutaneous lesions in patients with AIDS-related Kaposi’s sarcoma. Some PC-related proteins could be targeted by taking micronutrient supplements, such as Vitamin A for targeting PTGDS, Zinc chloride and Zinc sulfate for HRG, and Glutathione for HAGH. Furthermore, insulin targeting PCSK1 was widely used in treating diabetes mellitus and improving glycemic control.

### Phenome-wide MR analysis

A phenome-wide MR analysis regarding the causality of target proteins on 782 non-PC traits retrieved from UK Biobank was carried out to investigate the possible side effects and repurposing. Ultimately, a total of 51 causal relationships involving 12 proteins reached statistical significance, and no association was found for APOM, CFD, CHST9, FGFBP3, HAHG, LINGO1, MAN2B2, PTGDS, and STAMBP. Among the positive associations, over a half (28/51) owed to protein ABO, with the increased level of which mainly leading to thrombosis and cardiovascular events but preventing hemorrhage of digestive tract. Some other molecules also played a two-faced role in non-PC disease risk. For instance, targeting PCSK1 might benefit in alleviating cardiomegaly but elevate the risk of osteoarthrosis and other arthropathies. Additionally, of these plasma proteins in relation to non-PC phenotypes, interference on APOA5, HRG, and LIPN could be repurposed as treatment for some other digestive system illness with no prominent side effect. To note, reducing the plasma concentration of PDIA5 and CST5 might increase the susceptibility of stomach cancer and uterus cancer, respectively. The Additional file 1: Table S13 documented full results for other traits influenced by PC-related plasma proteins.

## Discussion

Early detection and valid treatment options for PC have long been a formidable challenge. To overcome this obstacle, increasing attention has been paid to the complex interaction of plasma proteins and cancers in recent years: on the one hand, the onset and development of tumors could be accompanied by alteration in concentration of circulating proteins due to the secretion by oncocytes and tumor-associated stromal and immune cells, and these proteins might serve as valuable biomarkers for diagnosis and prognosis prediction [15, 57, 58]; on the other hand, these proteins are involved in multiple process of carcinogenesis, tumor invasion, metastasis, and shaping the tumor microenvironment [59, 60]. Given the above facts, it is worthwhile to give a deep insight into the causal relationship between plasma protein and PC to assist efficient identification of potential diagnostic markers and interference targets by applying advanced analytic methodology with high-support evidence and less likelihood for confounding bias. With the emergence of the MR method, one previous research preliminarily attempted the causal inference of plasma protein levels and pan-cancer, including PC [61]. Nevertheless, the instruments used in that study were not cis-acting, increasing the risk of horizontal pleiotropy. Besides, external validation with additional data sources was not conducted, making the conclusion less persuasive. Furthermore, the role of cancer-specific risk factors in established protein-cancer correlation, as well as the possible side effects for therapies targeting these proteins were not well investigated. Hence, a more rigorous and comprehensive design was required.

In this study, after stringent quality control approaches, we obtained eligible cis-pQTLs that satisfied the basic assumptions of MR, and they were further utilized in the following proteome-wide MR analysis. In the primary proteome-wide investigation, genetically determined plasma concentration of 21 proteins was identified to significantly correlate with PC risk. Then the subsequent analyses could be summarized into three parts according to their analytic purpose. In part one analysis for validation, no reverse causation and horizontal pleiotropy were found for the 21 significant associations through bidirectional MR analysis, additional MR methods, repeated MR with varying IVs inclusion criteria, and other sensitivity analyses. The causal effect of ABO and CHST11 was confirmed by Bayesian colocalization with high-support evidence, while the effect of LINGO1 and PTGDS was confirmed with medium-support evidence. After conducting replication analysis with alternative datasets for exposure and outcomes, respectively, and after subsequent meta-analysis, the causality was no longer significant or inconsistent in direction with primary results for 10 proteins including PTGDS, MAN2B2, DPT, PDIA5, STAMBP, FGFBP3, LINGO1, APOA5, APOM, and XXYLT1, and they were categorized into tier 3 proteins with relatively low credibility. ABO and CHST11 lay in the tier 1 group on account of strong colocalization evidence while the rest of the other 9 proteins (CFD, ELANE, HAGH, CST5, CHST9, PSG5, PCSK1, HRG, and LIPN) were set as tier 2 category. In summary, the part one analyses successfully verified and underlined 11 plasma proteins (tier 1 and tier 2 proteins) in causal relation to PC with more convincing multi-dimension evidence. Subsequently, in part two analysis for exploration of the underlying pathways, we tested these identified associations at a transcription level in whole blood and pancreas tissues and looked into the differential expression of these protein-coding genes in specific cell clusters. Then the enrichment analysis, PPI networks, mutual causation, and animal knock-out models were also applied attempting to comprehend the biological significance and interaction of these targets. More importantly in part two analysis, the mediation analysis indicated the partial involvement of BMI and type 2 diabetes as mediators in the procancerous effect of CHST11 and ABO on PC, respectively. Finally, in the part three analysis for druggability, these targets were prioritized by searching drug-target databases and whether the risk of other diseases would be elevated when targeting the proteins for treatment was assessed through a phenome-wide MR analysis. To sum up, drugs had been developed for eight of the candidates, among which no evidence for prominent side effects was found upon targeting HRG, HAHG, and PTGDS, suggesting their promising potentials as safe therapeutic targets.

Protein ABO and CHST11 were causally linked to PC with the most convincing evidence in the present study. ABO (Histo-blood group ABO system transferase) is a glycosyltransferase enzyme participating in the biosynthesis of A and B antigens and determining the ABO blood type of individuals. In accordance with our findings, both epidemiological and genetic evidence have revealed a decreased susceptibility of PC in O blood type individuals in comparison to non-O groups [62, 63]. PC patients PC carrying O blood group also experienced more favorable survival [64]. The exact underlying mechanisms behind this connection remain unclear and are possibly ascribed to systemic inflammatory and immune response [65–67]. Interestingly, type 2 diabetes, a known risk factor for PC, was found to partially mediate the causal effect from ABO to PC in our study, despite the controversial association between blood group and diabetes in previous reports [68, 69]. Similarly, a retrospective study revealed a higher proportion of B blood type patients among those with long-term diabetes before PC diagnosis than that among PC patients without diabetes at diagnosis [70], which also implied the intricate interaction between ABO blood type, diabetes mellitus, and PC. However, although substantial evidence has demonstrated the strong link of ABO with PC, treatment targeting ABO is elusive and challenging considering the ambiguous impact on blood type and the multitude of side effects anticipated by our phenome-wide investigation (Additional file 1: Table S13). CHST11 (Carbohydrate sulfotransferase 11) as a member of the carbohydrate sulfotransferases family, is engaged in the modification of glycosaminoglycans, specifically in the sulfation of chondroitin sulfate, which is a crucial molecule in cancer progression and metastasis [71, 72]. And the microenvironment of PC tissue was observed enriched with chondroitin sulfate at a 22-fold increase in concentration compared to paired normal tissues [73]. It was also reported the high expression of CHST11 indicated poor prognosis for PC patients and correlated with worse clinical stage and histological grade [74]. Consistently, the pro-tumorigenesis effect of CHST11 was verified in this study, and a limited indirect effect through modulating BMI was additionally observed, throwing new insights into the possible interpretation of the association. Nevertheless, while experimental studies have shown CHST11 could drive cancer invasion, epithelial-mesenchymal transition, and cancer stem cell generation by activating signaling pathways such as Wnt/beta-catenin in multiple other types of malignancies [75, 76], its direct biological significance on PC cells has yet to be elucidated. Further laboratory evidence is required to confirm and expand our findings.

In addition to ABO and CHST11, significant correlations between another nine plasma proteins with PC were supported by external validation and meta-analysis, in spite of negative results from Bayesian colocalization. Noteworthily, there is still controversy on whether colocalization analysis violates the core MR assumptions, especially when utilizing quantitative trait loci as instruments, and negative colocalization results thereby do not necessarily attenuate the plausibility of the inferred causality [51, 77]. Of these candidate targets in tier 2, CFD, ELANE, and HAGH demonstrated the strongest statistical correlation with PC upon ordering the adjusted P-values (Table 1). CFD (Complement factor D) functions by enzymatically cleaving factor B when it is complexed with C3b in the alternative pathway of the complement system. Reports on the relationship between CFD and PC are limited, with only one retrospective study presenting that the CFD expression seemed to be irrelevant to the prognosis of PC patients undergoing neoadjuvant chemotherapy and surgery [78. However, regarding other tumors, laboratory experiments revealed that CFD stimulated proliferation in cutaneous squamous cell carcinoma by modulating ERK1/2 signaling pathway [79. In particular, CFD is also known as one of the obesity-driven biomarkers, and CFD along with its downstream effector hepatocyte growth factor secreted by adipocytes could augment the properties of cancer stem cells in breast cancer [80, 81]. Given that PC is another malignancy linked to obesity and adipose accumulation, future investigation into the role of CFD within the mechanisms through which obesity contributes to PC could be of vital value. ELANE (Neutrophil elastase) is a serine protease predominantly secreted by neutrophils and is implicated in the degradation of extracellular matrix proteins in the process of inflammation against pathogens [82]. It is also one of the essential components of neutrophil extracellular traps, which has been proven to activate pancreatic stellate cells to form a thick, fibrotic stroma and accelerate PC growth [83]. A previous report illuminated that ELANE played a mediator role in intratumoral bacteria and PC carcinogenesis, by shaping a pro-inflammatory tumor microenvironment [84]. In line with these findings, an elevated concentration of ELANE at a plasma protein or transcription level led to increased PC risk in our study. Besides, the declined secretion of tumor necrosis factor was highlighted after ELANE knock-out in mouse models. And drugs targeting ELANE have been approved in treating some inflammatory diseases, such as chronic obstructive pulmonary disorder. HAGH (Hydroxyacylglutathione hydrolase), also described as Glyoxalase II (GLO2), together with Glyoxalase I (GLO1) constitutes the glyoxalase system that is involved in the detoxification of methylglyoxal produced during the glycolytic pathway. In this study, the genetically determined plasma concentration of HAGH/GLO2 was positively associated with PC susceptibility. Consistently, numerous researches have implied the involvement of GLO1 and GLO2 in progression of multiple tumors. In PC, up-regulation of GLO1 was spotted in cancerous tissues and indicated poor outcome and acquired resistance to gemcitabine [85, 86]. In comparison, studies on GLO2 are scant and primarily focused on urological malignancies. For instance, GLO2 was observed to promote proliferation and elude apoptosis via mechanisms involving p53-p21 axis [87].

This study has a number of noticeable advantages. To the best of our knowledge, the current study presented the most extensive and comprehensive proteome-wide MR analysis for PC. The breadth, depth, and rigorousness of this study allowed for a more robust identification of promising targets for the development of screening biomarkers and therapeutic drugs for PC. First, numerous measures were taken to evade violation of basic MR assumptions and diminish the risk of confounding bias. We excluded pQTLs located within the MHC region or distant from the vicinity of the corresponding protein-coding gene (trans-pQTL). Considering MR analysis is susceptible to the IVs selection, we repeated our analysis with additional IVs inclusion criteria that has been employed in previous proteome-wide MR studies [88, 89]. A series of supplementary sensitivity analyses, reverse MR analysis, and colocalization analysis were conducted to enhance the robustness of identified associations. Consistent results from replicative MR analysis with replaced data source and subsequent meta-analysis, as well as MR or SMR using eQTL data of blood and pancreas tissues, also minimized the false positive risk of the conclusions. Second, evidence from enrichment analysis, PPI, mutual causation analysis, and single gene knock-out models, could provide potential views on how these candidate targets interact with PC. Third, we clarified a partial involvement of established PC risk factors, especially type 2 diabetes and BMI, in the pathogenic pathway of these plasma proteins. Last but not least, these identified targets were prioritized with druggability and side effects with three distinct drug-target databases and a phenome-wide MR investigation.

However, there are still several limitations. First, the enrolled populations in this study were mostly European individuals, and this restricted the expansion of our conclusions to other ancestries. Second, although employing cis-pQTLs instead of trans-pQTLs as instruments could avoid horizontal pleiotropy as much as possible, it would reduce the number of assessable candidates as a result of no eligible SNP for some proteins. To counteract this, we included up to six distinct proteomic studies in the primary stage, but it was still noteworthy that measurement bias might exist among these researches. Third, this study principally focused on the proteins in plasma, and their effects in pancreas tissues were not explored due to insufficient available data. Similarly, because of the lack of eligible eQTLs, investigation regarding the association at an expression level was unavailable for some of the candidate targets. Moreover, it was worth noting that the STRING database applied for PPI analysis was not PC tissues-specific, meaning these results should be interpreted more conservatively. Last, despite the superiority of MR approach in causality inference, it was rarely possible to thoroughly eliminate confounding bias or reverse causation. Consequently, large epidemiological and experimental studies are warranted to support the above results, and our plans are currently underway to gradually carry out cell experiments and animal experiments to offer further evidence for the pathogenic role of part of interested proteins.

In conclusion, a total of 21 plasma proteins were identified with etiological significance for PC. Two and nine of them were prioritized with the most convincing and medium-support evidence, respectively. With future effective validation, these candidate proteins might serve as novel biomarkers in PC early detection and promising druggable targets for PC treatment.

### Supplementary Information

Additional file 1 (XLSX 4422 KB)

## Data Availability

The data of FinnGen can be accessed at https://www.finngen.fi/en. The data of UK Biobank can be accessed at https://pheweb.org/UKB-SAIGE/. The GWAS summary statistics for PC based on a meta-analysis of the UK Biobank and GERA cohorts can be accessed at https://github.com/Wittelab/pancancer_pleiotropy. GWAS summary statistics for PC risk factors are available at UK Biobank (https://pheweb.org/UKB-SAIGE/) and IEU Open GWAS Project (https://gwas.mrcieu.ac.uk/). The pQTL data can be accessed at https://www.decode.com/summarydata/, https://www.synapse.org/#!Synapse:syn51364943/files/, https://gwas.mrcieu.ac.uk/, https://www.ebi.ac.uk/gwas/publications/37563310, and https://www.ebi.ac.uk/gwas/publications/33067605. The expression (eQTL) data for whole blood and pancreas tissue can be accessed at https://eqtlgen.org/phase1.html and https://yanglab.westlake.edu.cn/data/SMR/GTEx_V8_cis_eqtl_summary.html. The single-cell RNA sequencing data of PC tissue can be accessed at https://www.ncbi.nlm.nih.gov/geo/query/acc.cgi?acc=GSE155698.

## Abbreviations

PC: Pancreatic cancer
BMI: Body mass index
pQTL: Protein quantitative trait loci
Cis-pQTL: Cis-acting protein quantitative trait loci
MHC: Major histocompatibility complex
MAF: Minor allele frequency
eQTL: Expression quantitative trait loci
GWAS: Genome-wide association study
UKBPPP: UK Biobank Pharma Proteomics Project
MR: Mendelian randomization
SMR: Summary-data-based Mendelian randomization
HEIDI: Heterogeneity in independent instrument
LD: Linkage disequilibrium
IVs: Instrumental variables
SNP: Single nucleotide polymorphism
PPI: Protein–protein interaction
KEGG: Kyoto Encyclopedia of Genes and Genomes
STRING: Search Tool for the Retrieval of Interacting Genes
MGI: Mouse Genome Informatics
OR: Odds ratio
CI: Confidence interval
GEO: Gene Expression Omnibus
scRNA-seq: Single-cell RNA sequencing
